# Supplementary material for: Baseline Characterization of the Gut Microbiota of Field and Colony Populations of Phlebotomus tobbi and Preliminary Assessment of the Anti-Leishmanial Activity of Cultivable Bacteria
Source: Pathogens. 2026 Jun 23;15(7):658. doi: 10.3390/pathogens15070658 (PMC13416292; doi:10.3390/pathogens15070658)
Supplement: Supplementary file 1 [file pathogens-15-00658-s001.zip › pathogens-4357168-File S1/Taxa2ASV_Decomposer/Genus/g__Sphingomonas/seqs.mltree.pdf]

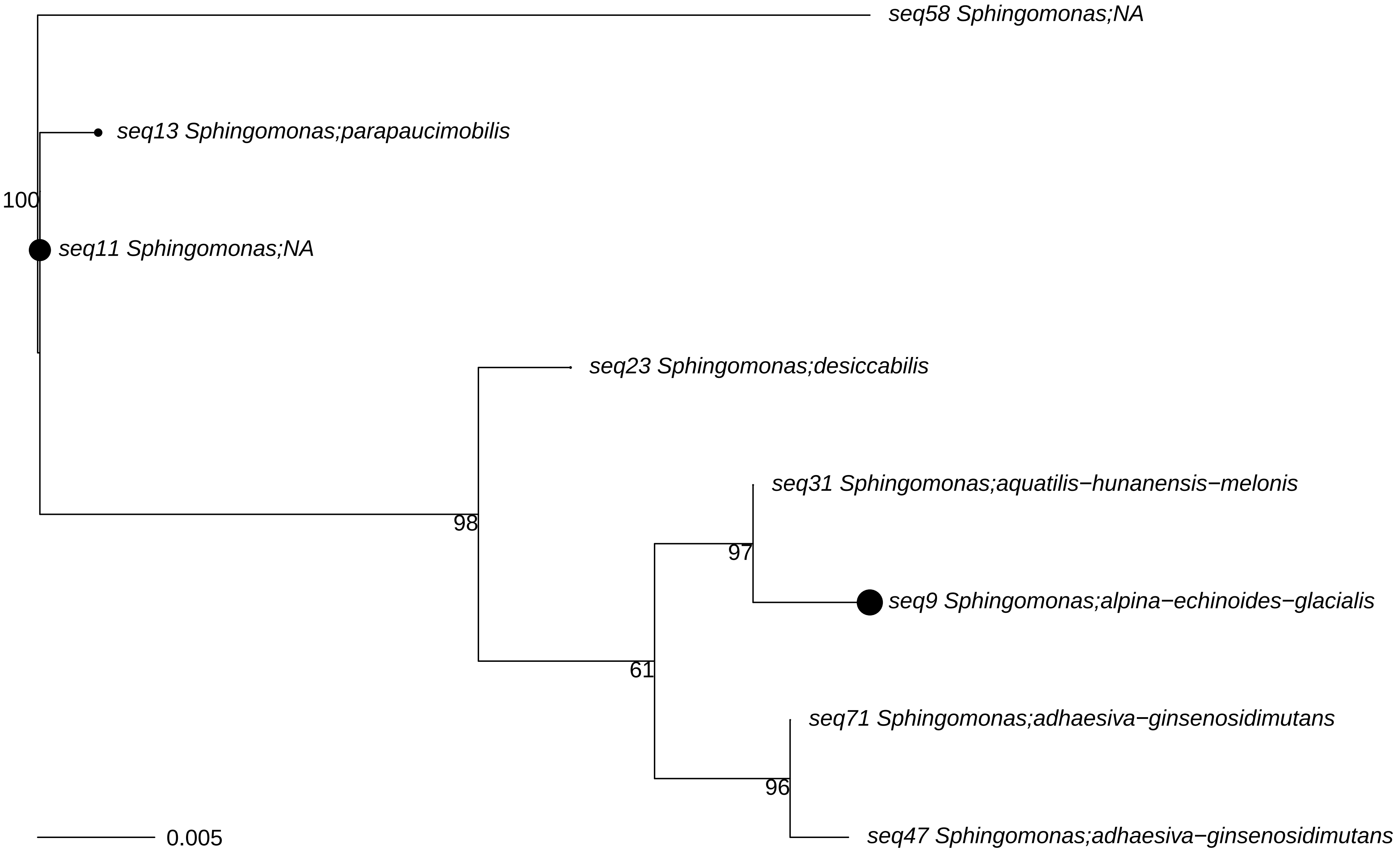
seq58 Sphingomonas;NA
seq13 Sphingomonas;parapaucimobilis
100
seq11 Sphingomonas;NA
seq23 Sphingomonas;desiccabilis
98
seq31 Sphingomonas;aquatilis−hunanensis−melonis
97
seq9 Sphingomonas;alpina−echinoides−glacialis
61
seq71 Sphingomonas;adhaesiva−ginsenosidimutans
96
seq47 Sphingomonas;adhaesiva−ginsenosidimutans
0.005
